# Determination of Urinary Hydroxyl PAHs Using Graphene Oxide@Diatomite Based Solid-Phase Extraction and High-Performance Liquid Chromatography

**DOI:** 10.3390/molecules24224186

**Published:** 2019-11-19

**Authors:** Yuanman Liu, Ziling Li, Ziyang Zhang, Tengwen Zhao, Manman Wang, Xuesheng Wang

**Affiliations:** School of Public Health, North China University of Science and Technology, Tangshan 063210, China; leo_mman@163.com (Y.L.); liziling02018@163.com (Z.L.); zzyang94@163.com (Z.Z.); ztwen_hbu@163.com (T.Z.)

**Keywords:** graphene oxide, diatomite, solid-phase extraction, hydroxyl polycyclic aromatic hydrocarbons, urine

## Abstract

A diatomite supported graphene oxide composite (GO@Dt–NH_2_) was fabricated and explored as a solid-phase extraction adsorbent coupled with high performance liquid chromatography to determine the trace hydroxyl polycyclic aromatic hydrocarbons (2-hydroxy-naphthalene, 2-hydroxy-fluorene, 1-hydroxy-phenanthrene, and 1-hydroxy-pyrene) in urine samples. The fabricated composites were characterized by X-ray powder diffractometry and scanning electron microscopy. GO@Dt–NH_2_ offered enhanced adsorption affinity towards the analytes compared with the bare diatomite. The amount of graphene oxide and the factors affecting solid-phase extraction were investigated in detail. Under the optimized conditions, the method gave good linearity (0.30–200 ng/mL) and a low detection limit (0.10–0.15 ng/mL) for the hydroxyl polycyclic aromatic hydrocarbons. The average recovery for spiked urine samples with three levels ranged from 90.6% to 100%. The intra-day and inter-day relative standard deviations were in the range of 1.8–6.4% and 2.7–11.8%, respectively. Besides, the GO@Dt–NH_2_ provided enrichment factors of 18–20 and superior purification ability. The developed method was successfully applied to the determination of hydroxyl polycyclic aromatic hydrocarbons in urine samples from smoking volunteers.

## 1. Introduction

Polycyclic aromatic hydrocarbons (PAHs) are a class of ubiquitous environmental contaminants originating from the incomplete combustion and pyrolysis of organic materials such as wood, fossil fuels, and tobacco [1]. Human exposure to these compounds occurs in multiple sources, including the environment (polluted air and drinking water), occupation (coke oven or aluminum and steel manufacture), personal habits (smoking), and diet (barbecue, roasting, broiling, or cooked foods) [2]. Chronic exposure to PAHs has been associated with lung cancer [3,4,5], peripheral arterial disease [6], female breast cancer [7], and colorectal cancer [8]. Given their wide distribution and their carcinogenic, mutagenic, and teratogenic toxicity, PAHs can pose potential risks to human health. However, the direct assessment of PAHs remains a great challenge because of their various entry routes. Hydroxylated PAHs (OH-PAHs), as metabolites of PAHs in urine, are commonly used as stable internal biomarkers to comprehensively estimate the total external PAH exposure level and health risk [9,10].

The measurement of OH-PAHs in urine is usually performed by gas chromatography-mass spectrometry (GC-MS) or high-performance liquid chromatography (HPLC) with MS or a fluorescence detector (FLD) [11,12,13,14,15,16,17,18,19,20,21,22]. However, the complexity of biological matrices and the trace concentrations of target analytes require a clean-up and enrichment technique to obtain satisfactory sensitivity and specificity of the method. Consequently, sample pretreatment approaches such as solid-phase extraction (SPE) [13,14], solid-phase microextraction (SPME) [15,16,17], magnetic solid-phase extraction (MSPE) [18,19,20], and online SPE [21,22] have been commonly employed before chromatographic determination. Among these sorption-based methodologies, including the modes of SPE, SPME, and MSPE, the adsorbent plays a key role in the extraction efficiency of the analytes of interest. Current improvements mainly focus on the development of novel adsorbents with enhanced recoveries, reduced cost, and improved compatibility with chromatographic techniques.

Graphene (G), is a newfangled class of intriguing carbon material with a two-dimensional honeycomb monolayer structure consisting of sp^2^ hybridized carbon atoms [23,24]. Graphene oxide (GO) is the oxidized derivative of graphene that has abundant highly reactive oxygen-containing functional groups on its basal plane or at the sheet edges. Their remarkable features, including their large surface area, extraordinary π delocalized electron system, and inexpensive synthesis process make G/GO powerful sorbents in the field of sample pretreatment [25,26,27,28,29,30].

However, the direct use of G/GO as an adsorbent by packing into a classical SPE cartridge has several disadvantages: First, small particles of G/GO can cause high pressure and miniscule G/GO sheets tend to escape from the packed cartridge, especially under high pressure; and second, the strong interplanar interactions of G/GO may lead to aggregation, and thus, the available surface area for adsorption is reduced dramatically. These problems may not only decrease the adsorption efficiency and reusability of the cartridge but also affect the accuracy and precision of the method. To avoid the above-mentioned obstacles while still taking advantage of these unique properties of G/GO, integrating G/GO with other supporting materials to fabricate composites or hybrids is a good strategy [31,32,33]. G/GO-bound silica adsorbents were developed by covalently binding G/GO sheets to silica via linkage of the carboxy groups of GO and the amino groups of an amino terminated silica. GO@silica was then reduced using hydrazine to obtain G@silica. In this way, G@silica and GO@silica were used to extract chlorophenols from environmental water samples and hydroxylated polybrominated diphenyl ethers from hexane solution, respectively [34]. G and titanium dioxide nanocomposites were integrated as an SPE sorbent for the purification and enrichment of phospholipids from avocado [35].

Diatomite (Dt) is a type of natural geological deposit composed of amorphous silica (SiO_2_·nH_2_O). It exhibits many unique properties including high porosity (80%–90%), good permeability, low density, and excellent thermal and chemical stability. Besides, Dt is abundant all over the world and readily available in large quantities (tons) at low cost. Compared with synthetic silica, Dt possesses the advantages of low cost and good biocompatibility [36]. Attributed to these advantages, Dt has been widely utilized as a catalyst carrier, filter agent, and adsorbent for contamination removal from wastewater [37]. For example, natural Dt has been reported as an effective pretreatment adsorbent for a nanofiltration system to adsorb fluoride from an aqueous solution [38]. In addition, Dt is commercially used as a solid–liquid extraction adsorbent for the pretreatment of polycyclic musks in blood and urine and *N*-nitrosamines in agricultural food [39,40]. Dt can also be used as a sort of feasible supporting material. Silicalite-1 nanoparticles were synthesized in situ on the surface of the modified Dt to form hierarchically porous Dt/silicalite-1 composite for benzene removal [41].

Herein, to overcome the main obstacles for the direct application of GO and benefit from the advantages of Dt, we report the synthesis of a novel composite, namely Dt-supported GO, and the application for SPE of OH-PAHs in urine. The as-synthesized composite was characterized by X-ray powder diffractometry (XRD) and a scanning electron microscope (SEM). The effect of GO and its amount in the GO@amino-terminated Dt (GO@Dt–NH_2_) composite on the adsorption performance for the four OH-PAHs was explored in detail. Parameters such as the loading volume, eluting solvent, and volume were optimized. Moreover, the GO@Dt–NH_2_ composite was applied for the SPE of OH-PAHs in urine samples.

## 2. Results and Discussion

### 2.1. Fabrication and Characterization of the GO@Dt–NH_2_ Composite

**Figure 1:**
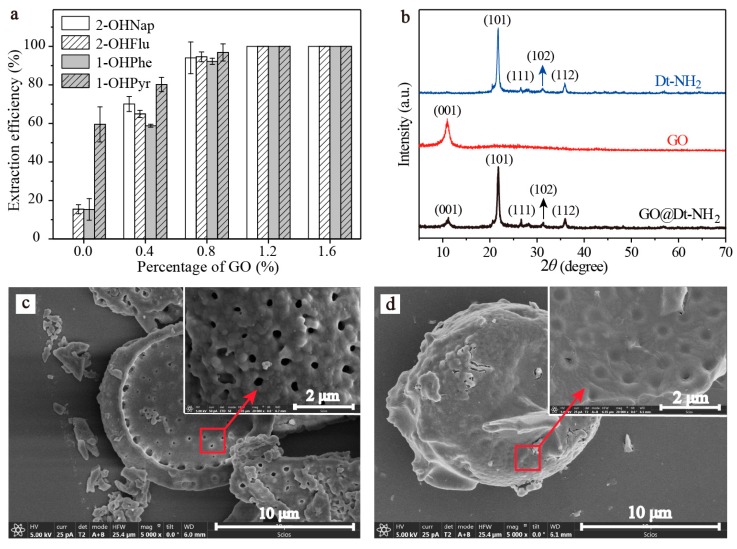
(**a**) Effect of percentage of GO (0.0 to 1.6%) in the reaction mixture on extraction efficiency, (**b**) XRD patterns of Dt–NH_2_, GO, and GO@Dt–NH_2,_ SEM images of (**c**) Dt–NH_2_ and (**d**) GO@Dt–NH_2_ (5000×, inlet, 20 000×).

In this study, GO nanosheets were developed to load on the Dt–NH_2_ via covalent coupling of the carboxy groups of GO and the amino groups of Dt–NH_2_ using N, N′-dicyclohexylcarbodiimide (DCC) as a coupling agent. 2-Hydroxy-naphthalene (2-OHNap), 2-hydroxy-fluorene (2-OHFlu), 1-hydroxy-phenanthrene (1-OHPhe), and 1-hydroxy-pyrene (1-OHPyr) were selected as testing analytes to investigate the effect of the mass percentage of GO in the reaction mixture (0.0%, 0.4%, 0.8%, 1.2% and 1.6%) on the extraction efficiency (Figure 1a). To guarantee the complete adsorption equilibrium, 100 mg of each synthesized composite was added to 2.0 mL of a 20 ng/mL spiked aqueous sample solution. The adsorption efficiency was evaluated by the difference in the peak areas of OH-PAH standard solutions and that detected in the effluents of GO@Dt–NH_2_. As shown in Figure 1a, the adsorption efficiencies of Dt–NH_2_ for OH-PAHs were in the range of 0–59.5%. The increase in the mass percentage of GO in the reaction mixture from 0.4% to 1.6% dramatically enhanced the extraction performance of all the analytes and reached the maximum (100% for each analyte) when the percentage of GO exceeded 1.2%. It also definitely revealed the presence of GO in the as-prepared composites. Furthermore, 1.2% of the GO in the reaction mixture provided adequate adsorption for each analyte, significantly confirming the superiority of the hybrid GO and Dt–NH_2_ adsorbent. Thus, 1.2% of the mass percentage of GO in the reaction mixture was selected to prepare the GO@Dt–NH_2_ composites.

The prepared Dt–NH_2_, GO, and GO@Dt–NH_2_ were investigated by XRD measurements. As shown in Figure 1b, for GO@Dt–NH_2_, the existence of the typical peak of GO at 10.2° (001) and the appearance of the characteristic peaks of Dt–NH_2_ at 21.8° (101), 28.3° (111), 31.3° (102), and 35.9° (112) further confirmed the successful combination of GO and Dt–NH_2_. The morphology of the synthesized Dt–NH_2_ and GO@Dt–NH_2_ composites (7.5 mg GO) was characterized by SEM. SEM images (Figure 1c) indicated Dt–NH_2_ with a characteristic disk-like shape. Regularly spaced rows of pores (~300–500 nm in diameter) located along the frustule shell wall were clearly observed, indicating the porosity and large specific surface area of Dt. For GO@Dt–NH_2_ (Figure 1d), the Dt–NH_2_ was encapsulated completely by GO sheets. The SEM images demonstrate that GO sheets were immobilized onto the Dt–NH_2_ surface, as indicated by the arrows.

The adsorption capacities of Dt–NH_2_ and GO@Dt–NH_2_ for the OH-PAHs are listed in Table 1. Dt–NH_2_ gave adsorption capacities of 0.0–17.8 mg/g and the GO@Dt–NH_2_ provided greatly enhanced adsorption capacities ranging from 181.8 to 409.6 mg/g. In addition, the adsorption ability of GO@Dt–NH_2_ for the selected compounds followed an increasing order of their octanol–water partition coefficient (logP) values (logP_2-OHNap_ > logP_2-OHFlu_ > logP_1-OHPhe_ > logP_1-OHPyr_) and their numbers of benzene rings. These results likely resulted from the hydrophobic effect and π-π interactions between the GO and OH-PAHs.

### 2.2. Optimization of the SPE Conditions

In order to optimize the SPE conditions for OH-PAHs, several parameters, including the loading volume, eluting solvent, and its volume were investigated in detail. All of the optimization experiments were performed in triplicate.

#### 2.2.1. The Loading Volume

In this work, 100 mg of GO@Dt–NH_2_ adsorbent was selected to load the sample solution into the cartridge. To facilitate the pretreatment process and obtain high sensitivity, the loading volume was a crucial factor that influenced the extraction capacity and enrichment ability.

**Figure 2:**
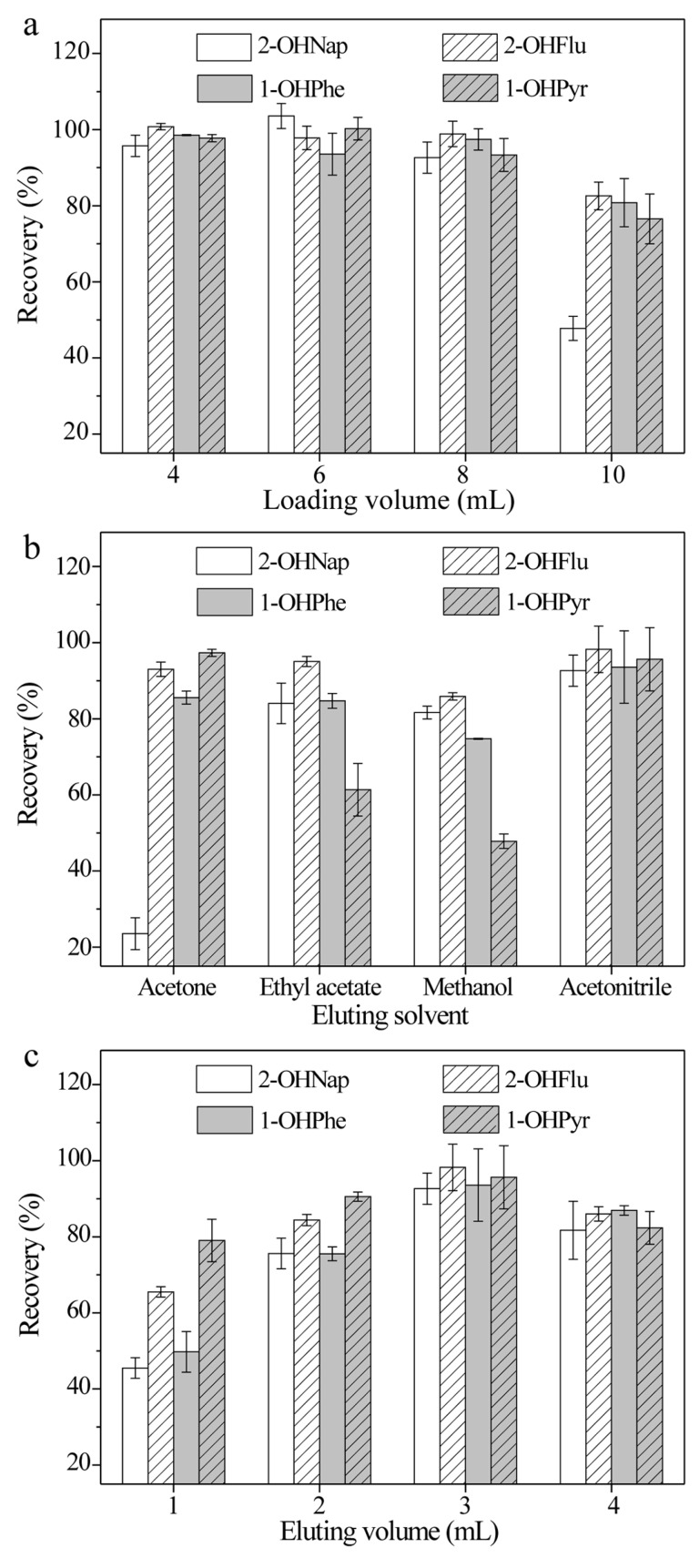
Effect of (**a**) the loading volume, (**b**) the eluting solvent, and (**c**) the eluting volume on the extraction efficiency of four OH-PAHs (*n* = 3).

Different loading volumes from 4 to 10 mL were investigated, with the constant spiked OH-PAH amount in loading samples. The adsorbed analytes were eluted using 3 mL of ACN. As shown in Figure 2a, recoveries ranging from 92.6% to 104% were obtained for loading volumes up to 8 mL, and further increases in the loading volume decreased the analyte recoveries to 47.8–82.6%. Considering the recovery and the sensitivity of the proposed method, a sample volume of 8 mL was selected for further work.

#### 2.2.2. Eluting Solvent and Eluting Volume

The elution process must make sure all analytes are desorbed from the sorbent with minimal carryover. Thus, the eluting solvent and its volume have a significant effect on the recovery of the method. Various elution solvents, including acetone, ethyl acetate, methanol (MeOH), and acetonitrile (ACN), were studied. Figure 2b indicates that ACN exhibited the best elution ability (recovery: 92.6–98.2%). A proper elution volume guarantees adequate desorption of the analytes from the adsorbent with minimum consumption of the solvent. The volume of elution solvent from 1 to 4 mL was also studied. Figure 2c shows that the recovery of OH-PAHs increased as the volume increased from 1 to 3 mL. An elution volume of 4 mL caused a slight decrease in the recovery of OH-PAHs. A large eluting volume is unfavorable as it extends the time for nitrogen (N_2_) evaporation which would also cause a loss of OH-PAHs. Thus, 3 mL of ACN was employed for the elution of OH-PAHs.

### 2.3. Validation of the GO@Dt–NH_2_ Based SPE HPLC-FLD Method

The analytical evaluation parameters including the linear range, correlation coefficients (*r*), limits of detection (LODs), limits of quantification (LOQs), accuracy, and precision were studied under the optimized experimental conditions. The results are listed in Table 2. The calibration curves were constructed using OH-PAHs at seven concentration levels from 0.30 to 200 ng/mL. For each concentration level, three replicate extractions and determinations were performed. As shown in Table 2, good linearity (*r* = 0.999) was obtained in the range of 0.50–200 ng/mL for 2-OHNap, 0.30–150 ng/mL for 2-OHFlu and 1-OHPhe, and 0.40–200 ng/mL for 1-OHPyr. The LOD (S/N = 3) was found to be 0.15 ng/mL for 2-OHNap, 0.10 ng/mL for 2-OHFlu and 1-OHPhe, and 0.12 ng/mL for 1-OHPyr, respectively. The LOQ (S/N = 10) in urine was found to be 0.50 ng/mL for 2-OHNap, 0.30 ng/mL for 2-OHFlu and 1-OHPhe, and 0.40 ng/mL for 1-OHPyr, which shows that the present method is sufficiently sensitive to monitor four OH-PAHs. Moreover, the enrichment factor (EF) was used to evaluate the enrichment ability of the method, which is defined as the ratio of the calibration curve slope of the analyte performing via SPE to that without SPE. The EFs of the four OH-PAHs were in the range of 18–20.

The results were further confirmed by the recovery experiments. The accuracy was evaluated using the recovery of the proposed method which was assayed on spiked urine samples at three analyte levels (0.5, 1, and 2 ng/mL). The precision was described based on the intra-day and inter-day relative standard deviations (RSDs). As shown in Table 2, the average recoveries of four OH-PAHs were in the range of 90.6–100%. The intra-day and inter-day RSDs were in the range of 1.8–6.4% and 2.7–11.8%, respectively. The reproducibility of the material was evaluated by calculating the RSDs of peak areas of OH-PAHs. The column-to-column RSDs and batch-to-batch RSDs for three parallel varied from 3.9–8.7% and 7.0–14.5%, respectively. The GO@Dt–NH_2_ adsorbents not only offer excellent extraction ability for OH-PAHs but also provide good precision and reproducibility. In conclusion, these results highlight the potential feasibility of GO@Dt–NH_2_ for the SPE of OH-PAHs in urine.

### 2.4. Urine Sample Analysis and Comparison with Commercial C_18_ Adsorbent

The proposed method was further applied for the analysis of OH-PAHs in urine samples collected from ten smokers. As indicated in Table 3, the concentration of 1-OHPhe was found to be 1.37 ± 0.08 ng/mL in only one sample, 2-OHFlu was detected in five cases ranging from 1.04 ± 0.07 to 3.18 ± 0.04 ng/mL. The concentration of 2-OHNap was found to be 0.91 ± 0.26 to 4.02 ± 0.08 ng/mL in nine samples and 1-OHPyr was detected in nine cases with a concentration of 1.23 ± 0.15 to 4.80 ± 0.01 ng/mL. The recoveries of the OH-PAHs obtained by spiking the 2 ng/mL standards in the urine samples ranged from 86.4% to 108%.

**Figure 3:**
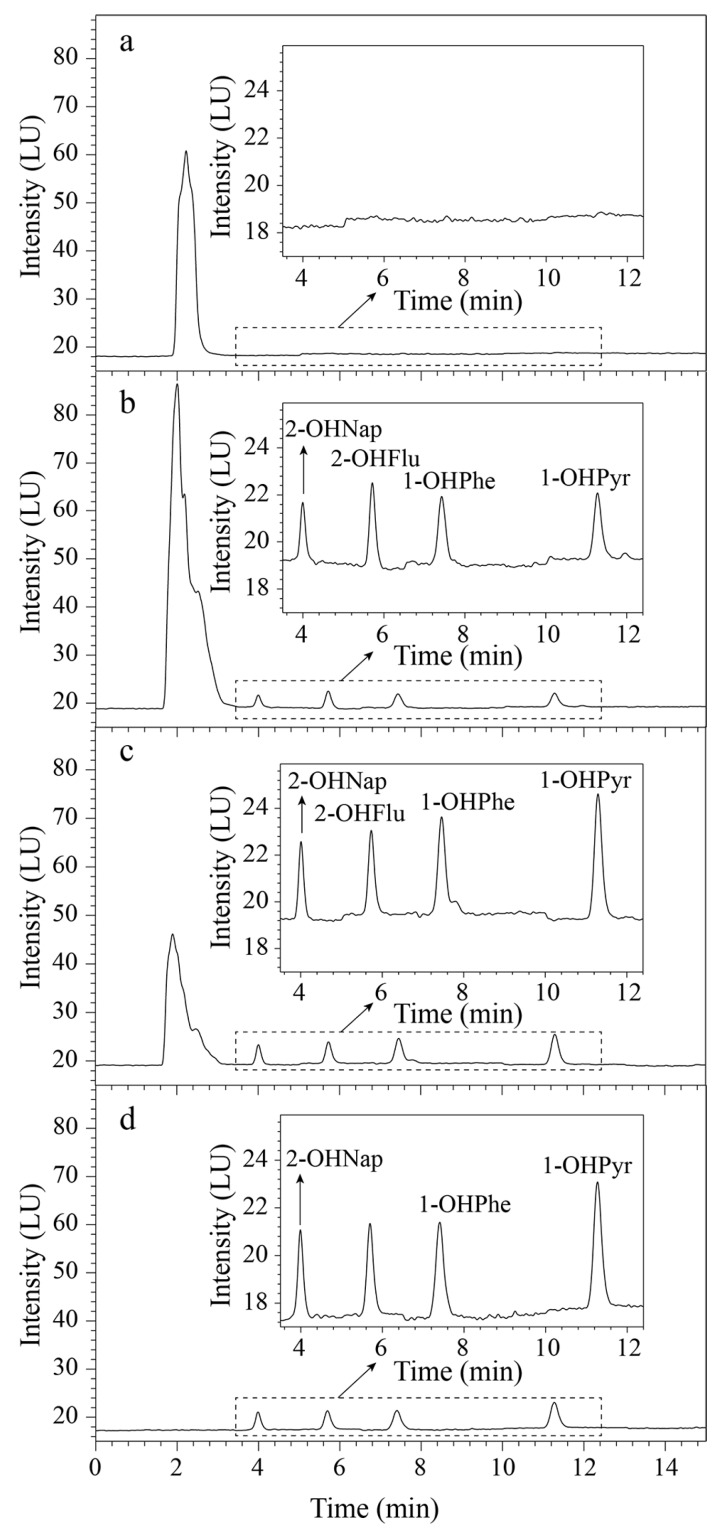
Chromatograms of urine samples (spiked with 2 ng/mL of 2-OHNap and 1-OHPyr, 1 ng/mL of 2-OHFlu and 2-OHPhe) with (**a**) direct injection, (**b**) solid-phase extraction (SPE) by commercial C_18_ adsorbent, (**c**) SPE of the GO@Dt–NH_2_ composites, and (**d**) the standard solution of OH-PAHs.

Figure 3a,c,d show the chromatograms of the urine sample without any SPE, the sample pretreated by the GO@Dt–NH_2_ cartridge, and the standard solution of the analytes. Four OH-PAHs were successfully purified and concentrated by the GO@Dt–NH_2_ adsorbent from complex urine samples. These results indicate the availability of the developed method.

Besides, the extraction performance of the GO@Dt–NH_2_ adsorbent was compared with that of the commercial C_18_ adsorbent. The evaluation employed 100 mg of each adsorbent with 8 mL of the urine sample solution spiked with 2 ng/mL of 2-OHNap and 1-OHPyr and 1 ng/mL of 2-OHFlu and 1-OHPhe. As shown in Figure 3b,c, both sorbents enable the purification and enrichment of the analytes from the urine sample. The interfering chromatographic peaks appear in the range of 1.5–3.0 min. Their intensities in Figure 3c are obviously lower than those shown in Figure 3b, indicating the superior removal ability of the GO@Dt–NH_2_ adsorbent for the interfering molecules. Moreover, the recoveries of four OH-PAHs obtained by commercial C_18_ adsorbent were 59.3–92.3% while the corresponding results were 89.0–98.4% using the GO@Dt–NH_2_ adsorbent. In addition, the starting material of natural Dt for the preparation of the GO@Dt–NH_2_ sorbent is readily available and rather inexpensive. These comparisons sufficiently confirmed that GO@Dt–NH_2_ has great advantages as an effective and economical adsorbent.

## 3. Materials and Methods

### 3.1. Chemicals and Standards

All reagents used were of analytical grade unless otherwise stated. GO was purchased from the Institute of Coal Chemistry, Chinese Academy of Sciences (Taiyuan, China). Dt (99.95%, particle size ≤ 30.8 μm), 3-aminopropyl-trimethoxysilane (APTES), N, N′-dicyclohexylcarbodiimide (DCC), and ethyl acetate were obtained from Aladdin (Shanghai, China). N, N-Dimethylformamide (DMF), acetic acid (HAc), and acetic acid sodium (NaAc) were provided by Tianjin Guang Fu Fine Chemical Research Institute (Tianjin, China). Acetone, hydrochloric acid (HCl), and ethanol were provided by Kermer Chemical Company (Tianjin, China). *β*-Glucuronidase/arylsulfatase from *HELIX POMATIA* (Type HP-2, aqueous solution, ≥100000 unites/mL) was purchased from Sigma-Aldrich (Milan, Italy). HPLC-grade MeOH and ACN were obtained from Sigma-Aldrich (Milan, Italy). Ultrapure water was provided by a Milli-Q water purification system (Millipore, Burlington, MA, USA). The empty SPE cartridges (polypropylene, 6 mL) were obtained from Bonna-Agela (Tianjin, China). ProElut C_18_ (100 mg) was purchased from DIKMA Technologies Inc. (Beijing, China).

2-OHNap (99.5%) and 2-OHFlu (98.0%) were purchased from Aladdin (Shanghai, China). 1-OHPhe (98.0%) and 1-OHPyr (99.8%) were purchased from Dr. Ehrenstorfer (Augsburg, Germany).

Stock solutions of standards (1.0 mg/mL for each) were prepared by dissolving four OH-PAHs with MeOH and kept at 4 °C in the dark. Working solutions were prepared daily by diluting the standard solution with MeOH.

### 3.2. Instruments

Chromatographic analysis was performed on an Agilent 1200 HPLC (Santa Clara, CA, USA), consisting of a G1322A degasser, a G1311A pump system, a G1329A autosampler, a G1316A temperature control center, and a G1321A FLD. The XRD patterns were acquired on a D/Max 2500 PC single crystal X-ray diffractometer (Rigaku, Akishima, Tokyo, Japan). SEM images of the prepared composites were obtained by a FEI JEM-2800F dual beam focused ion beam/field emission scanning electron microscope (Hillsboro, TX, USA).

### 3.3. Preparation of GO@Dt–NH_2_ Composites

Dt–NH_2_ was synthesized in accordance with Jiang et al. [42] with small modifications. Dt particles were initially treated with 2 mol/L HCl solution for 1 h and then washed thoroughly with ultrapure water and dried. The collected Dt (1 g) was mixed into 100 mL ethanol solution containing 1 mL APTES by sonication for 30 min to form a homogeneous suspension. After stirring at 60 °C for 8 h, the solids were collected by centrifugation and washed with ultrapure water and ethanol several times. The obtained Dt–NH_2_ product was dried under a vacuum at 60 °C for 12 h.

**Figure 4:**
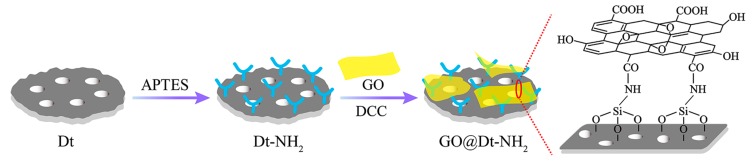
Schematic illustration of the fabrication of GO@Dt–NH_2_ composites.

GO@Dt–NH_2_ composites were prepared via the covalent coupling of the amino groups of Dt–NH_2_ and the carboxy groups of GO using DCC as a coupling agent. Typically, 7.5 mg of GO was dispersed in 100 mL of DMF under 1 h ultrasonication. Then, 500 mg of Dt–NH_2_ and 105 mg of DCC were added. The mixture was stirred at 50 °C for 24 h. The obtained gray slurry was washed with ultrapure water and MeOH repeatedly, and finally dried in a vacuum oven at 80 °C for 12 h. Figure 4 indicates a schematic illustration of the fabrication of GO@Dt–NH_2_ composites.

### 3.4. Urine Sample Collection and Preparation

Human urine samples (15 mL) were collected in the morning from 10 smoking volunteers. All volunteers signed informed consent forms for this study. This study was approved by the Ethics Committee of North China University of Science and Technology (Tangshan, China). All samples were frozen and stored at −80 °C until further analysis.

Prior to enzymatic hydrolysis, the urine sample was thawed at room temperature and briefly agitated. Five milliliters of urine was transferred into a 15 mL polypropylene centrifuge tube, and 5 mL of sodium acetate buffer (0.5 mol/L, pH = 5.0) and 10 μL of *β*-glucuronidase/arylsulfatase enzyme were added. The mixture was incubated for 12 h at 37 °C to release OH-PAHs from the conjugated forms. Finally, the resulting sample was centrifuged at 1500 rpm for 10 min. The supernatant was collected and stored at 4 °C.

### 3.5. SPE Procedure

The SPE cartridges were obtained by packing 100 mg of GO@Dt–NH_2_ adsorbents in 6 mL empty cartridges using polypropylene upper and lower frits to avoid adsorbent loss. For SPE of OH-PAHs from urine, the GO@Dt–NH_2_ cartridge was preconditioned with 2 mL of ACN and 2 mL of water. Eight milliliters of urine hydrolysate was loaded on the cartridge at a flow rate of 0.2 mL/min. The cartridge was kept in a vacuum for 5 min to remove the residual solvent. Subsequently, the cartridge was eluted with 3 mL ACN at a flow rate of 0.2 mL/min. The eluent was collected and evaporated to dryness under N_2_ steam. Finally, the residues were re-dissolved in 200 μL MeOH for subsequent HPLC analysis. Each extraction was carried out for three replicates for parallel testing.

### 3.6. HPLC-FLD Analysis

The chromatographic separation of OH-PAHs was performed on a polycyclic aromatic hydrocarbon (PAH) column (250 mm × 4.6 mm, 5 μm, Supelco, USA) while the following ACN–water gradient elution was applied: 0–5 min at 55% ACN, 5–13 min at a linear gradient from 55% to 85% ACN. The flow rate of the mobile phase was set to 1.0 mL/min. The sample injection volume was 20 μL, and the temperature was maintained at 20 °C. The following fluorescence Excitation (Ex)/Emission (Em) wavelength switching program was performed: 0–5 min at 227/355 nm (2-OHNap), 5–6.5 min at 272/366 nm (2-OHFlu), 6.5–10 min at 284/383 nm (1-OHPhe), and 10–13 min at 242/396 nm (1-OHPyr).

## 4. Conclusions

A novel GO@Dt–NH_2_ adsorbent was fabricated and applied for the SPE of OH-PAHs in urine samples with wide linear ranges, low detection limits, and satisfactory recoveries. Compared with the bare Dt–NH_2_ particles, the adsorption affinity of the developed GO@Dt–NH_2_ sorbent obtained for OH-PAHs was significantly enhanced. GO@Dt–NH_2_ sorbent obtained with a minimax ratio of 1.2% of GO in the polymerization provided an analyte enrichment factor of 18–20. Besides, the sorbent offered a superior purification ability compared with commercial C_18_ adsorbent. GO@Dt–NH_2_ composites are promising as effective and economical adsorbents for the SPE of trace analytes from biological samples.

## Figures and Tables

**Table 1:** The structure and logP values of the OH-PAHs and the adsorption capacities of Dt–NH_2_ and GO@Dt–NH_2._

Analyte	Structure	logP	Adsorption Capacity (mg/g)	
			Dt–NH_2_	GO@Dt–NH_2_
2-OHNap	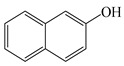	2.71	0.0	181.8
2-OHFlu	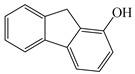	3.43	9.3	265.4
1-OHPhe	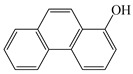	3.94	9.9	288.7
1-OHPyr	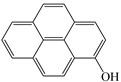	4.29	17.8	409.6

**Table 2:** Linear Range, Limits of Detection (LODs), Limits of Quantification (LOQ), Recovery, and Precision of the Developed Method.

Analyte	Linear Range (ng/mL)	Regression Equation ^1^ (*r*)	LOD (ng/mL)	LOQ (ng/mL)	Spiked Level (ng/mL)	Recovery (%)	Precision (RSD, %, *n* = 3)	
							Intra-day	Inter-day
2-OHNap	0.50–200	y = 0.431x + 2.17 (0.999)	0.15	0.50	0.5	95.5	6.4	11.8
					1	90.6	2.9	3.0
					2	93.9	4.3	11.3
2-OHFlu	0.30–150	y = 1.23x + 5.65 (0.999)	0.10	0.30	0.5	93.0	3.3	9.6
					1	95.0	4.3	5.7
					2	93.1	2.9	6.7
1-OHPhe	0.30–150	y = 2.92x + 7.76 (0.999)	0.10	0.30	0.5	93.2	6.1	8.6
					1	96.2	2.4	2.7
					2	100	5.6	3.7
1-OHPyr	0.40–200	y = 22.7x − 31.2 (0.999)	0.12	0.40	0.5	93.8	5.5	5.9
					1	95.5	2.7	7.9
					2	94.6	1.8	9.0

^1^ x, mass concentration, ng/mL, y, peak area.

**Table 3:** Analytical results for the determination of Hydroxylated PAHs (OH-PAHs) in urine samples from 10 smokers (*n* = 3).

Sample	OH-PAHs	Found ± SD (ng/mL)	Recovery ^1^ (%)	RSD (%, *n* = 3)	Sample	OH-PAHs	Found ± SD (ng/mL)	Recovery (%)	RSD (%, *n* = 3)
1	2-OHNap	2.16 ± 0.07	102	9.7	6	2-OHNap	N.D.	93.1	8.5
	2-OHFlu	1.04 ± 0.07	99.3	2.0		2-OHFlu	N.D.	95.3	3.1
	1-OHPhe	N.D. ^2^	93.3	7.7		1-OHPhe	N.D.	94.8	4.6
	1-OHPyr	4.60 ± 0.06	103	7.9		1-OHPyr	4.07 ± 0.15	96.2	7.5
2	2-OHNap	2.65 ± 0.26	96.6	2.1	7	2-OHNap	2.41 ± 0.07	98.1	10.7
	2-OHFlu	N.D.	97.7	4.4		2-OHFlu	1.14 ± 0.05	94.6	2.0
	1-OHPhe	N.D.	93.8	2.6		1-OHPhe	N.D.	93.8	5.6
	1-OHPyr	1.23 ± 0.15	95.0	6.0		1-OHPyr	4.47 ± 0.04	93.6	6.3
3	2-OHNap	0.91 ± 0.26	105	5.1	8	2-OHNap	2.78 ± 0.09	97.4	7.2
	2-OHFlu	N.D.	94.8	3.7		2-OHFlu	N.D.	96.9	4.3
	1-OHPhe	N.D.	100	2.5		1-OHPhe	N.D.	92.8	6.5
	1-OHPyr	2.02 ± 0.06	103	9.1		1-OHPyr	4.80 ± 0.01	86.4	7.6
4	2-OHNap	3.66 ± 0.15	105	5.6	9	2-OHNap	3.71 ± 0.11	100	5.7
	2-OHFlu	3.18 ± 0.04	88.0	3.3		2-OHFlu	N.D.	96.1	3.4
	1-OHPhe	N.D.	89.6	3.1		1-OHPhe	N.D.	96.1	3.6
	1-OHPyr	N.D.	92.9	4.7		1-OHPyr	2.70 ± 0.03	88.3	5.1
5	2-OHNap	4.02 ± 0.08	96.2	6.7	10	2-OHNap	2.88 ± 0.14	108	8.0
	2-OHFlu	1.33 ± 0.09	89.1	3.4		2-OHFlu	1.50 ± 0.11	87.0	4.0
	1-OHPhe	1.37 ± 0.08	101	3.8		1-OHPhe	N.D.	98.0	4.2
	1-OHPyr	2.22 ± 0.07	96.8	2.7		1-OHPyr	3.66 ± 0.09	90.1	3.8

^1^ Recovery data for spiked 2 ng/mL for each analyte. ^2^ N.D., Not detected.
